# Supplementary material for: ZFYVE21 is a complement-induced Rab5 effector that activates non-canonical NF-κB via phosphoinosotide remodeling of endosomes
Source: Nat Commun. 2019 May 21;10:2247. doi: 10.1038/s41467-019-10041-2 (PMC6529429; doi:10.1038/s41467-019-10041-2)
Supplement: Supplementary file 1 — Supplementary Information [file 41467_2019_10041_MOESM1_ESM.doc]

**
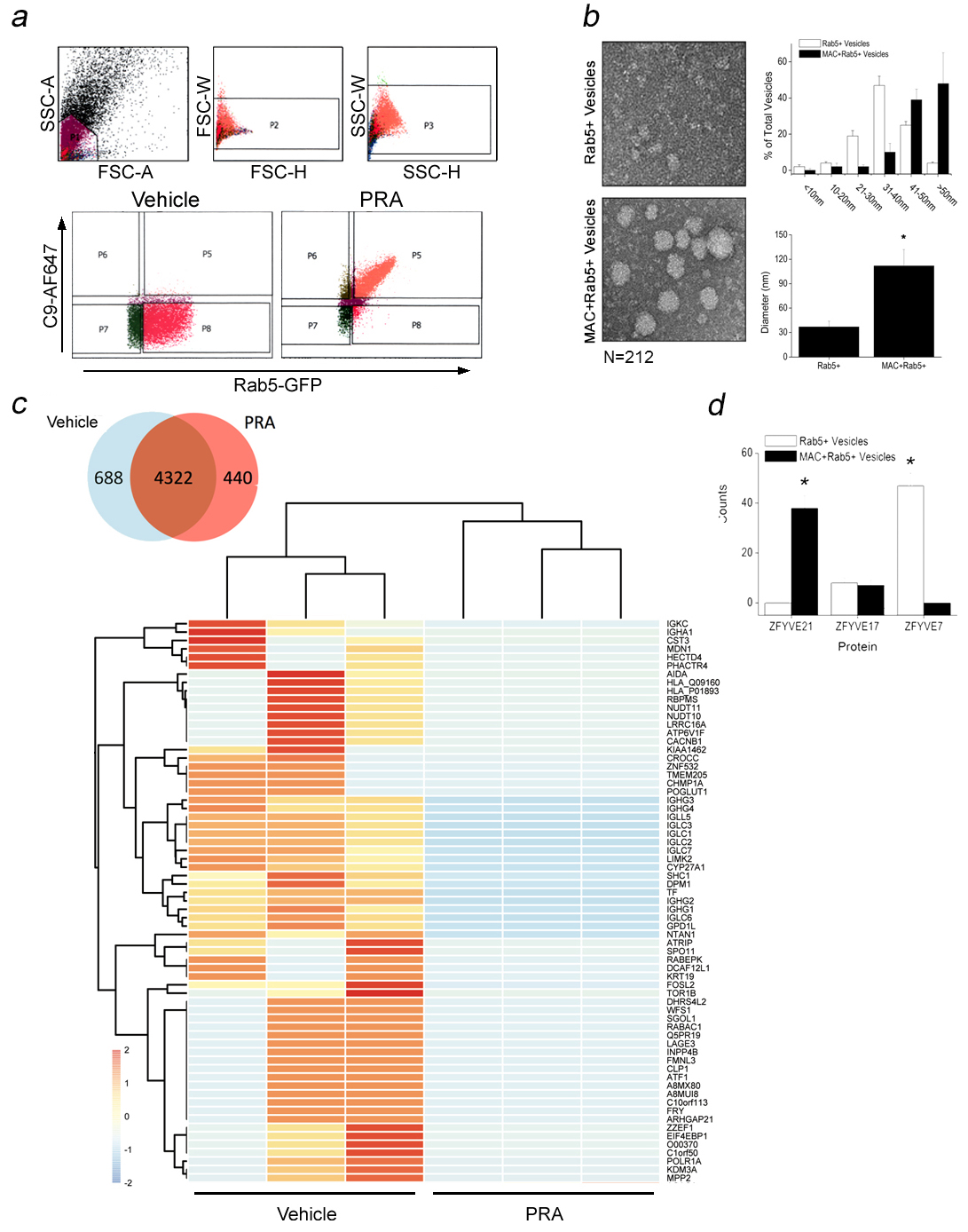
**

**Supplementary Figure 1. Proteomic Analysis of FACS-Sorted MAC+Rab5+ Endosomes.** Rab5-GFP HUVEC were treated with PRA sera containing C9-AF647. Forty-five minutes after treatment, HUVEC were sonicated and vesicles were gated and sorted by FACS. Rab5-GFP+ events in untreated HUVEC were used as controls (*a*). Diameters of single-membraned vesicles were measured in PRA-treated HUVEC *in situ* using immune-electron microscopy against C9 (arrows) and Rab5 (*b,* arrowheads, scale bar: 800μm). FACS sorted Rab5+ vesicles from untreated HUVEC and MAC+Rab5+ vesicles from PRA-treated HUVEC were analyzed by electron microscopy (*c*). Absorbance of C9 AF647 protein products was assessed prior to and following ultracentrifugation (*d*, left). Percent change (*d*, middle) and absolute change (*d,* right) of absorbance were calculated. Prior to (C9 AF647 Pre) and following (C9 AF647 Post) ultracentrifugation, C9 AF647 protein products were used to treated HUVEC (25% v/v in gelatin veronal buffer) for 30 min (*e*). Venn diagram and heat map of vesicular proteins (*f*). Spectral counts of FYVE domain proteins identified in the analysis (*g*). Proteomic analyses were repeated three times using three separate HUVEC donors. Representative data shown.

**
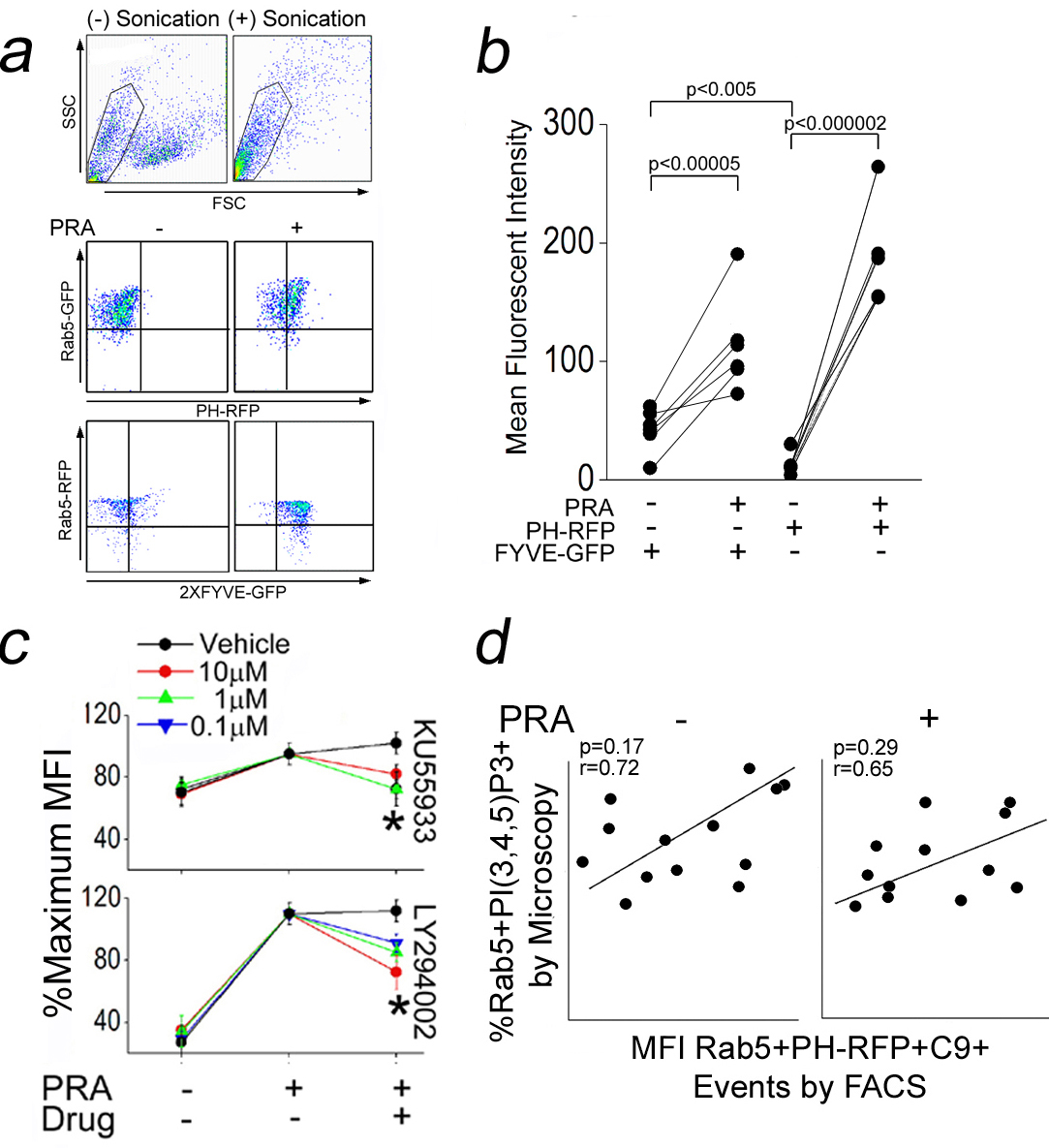
**

**Supplementary Figure 2. FACS-Assisted Assay for Assessing Vesicular Phosphoinositides.** Rab5 reporter HUVEC were co-transduced with either FYVE-GFP or PH-RFP and treated with PRA sera containing C9 AF647. Subcellular contents were released by sonication, gated, and MFIs of C9+Rab5+FYVE+ and C9+Rab5+PH+ MFIs were assessed (*a*). PRA treatment increased C9+Rab5+FYVE+ and C9+Rab5+PH+ MFIs (*b*). Dose-dependent pharmacologic depletion of PI(3)P with KU55933 (*c*, top, n=3) or PI(3,4,5)P3 with LY294002 (*c*, bottom, n=3) reduced reporter MFIs. Correlations between PI(3)P and PI(3,4,5)P3 reporters with confocal microscopy staining (*d*). Lipid reporter assays were repeated three times using 8 technical replicates per group per experiment. Correlations between lipid reporters and confocal microscopy were assessed using 12-15 technical replicates per group.

**
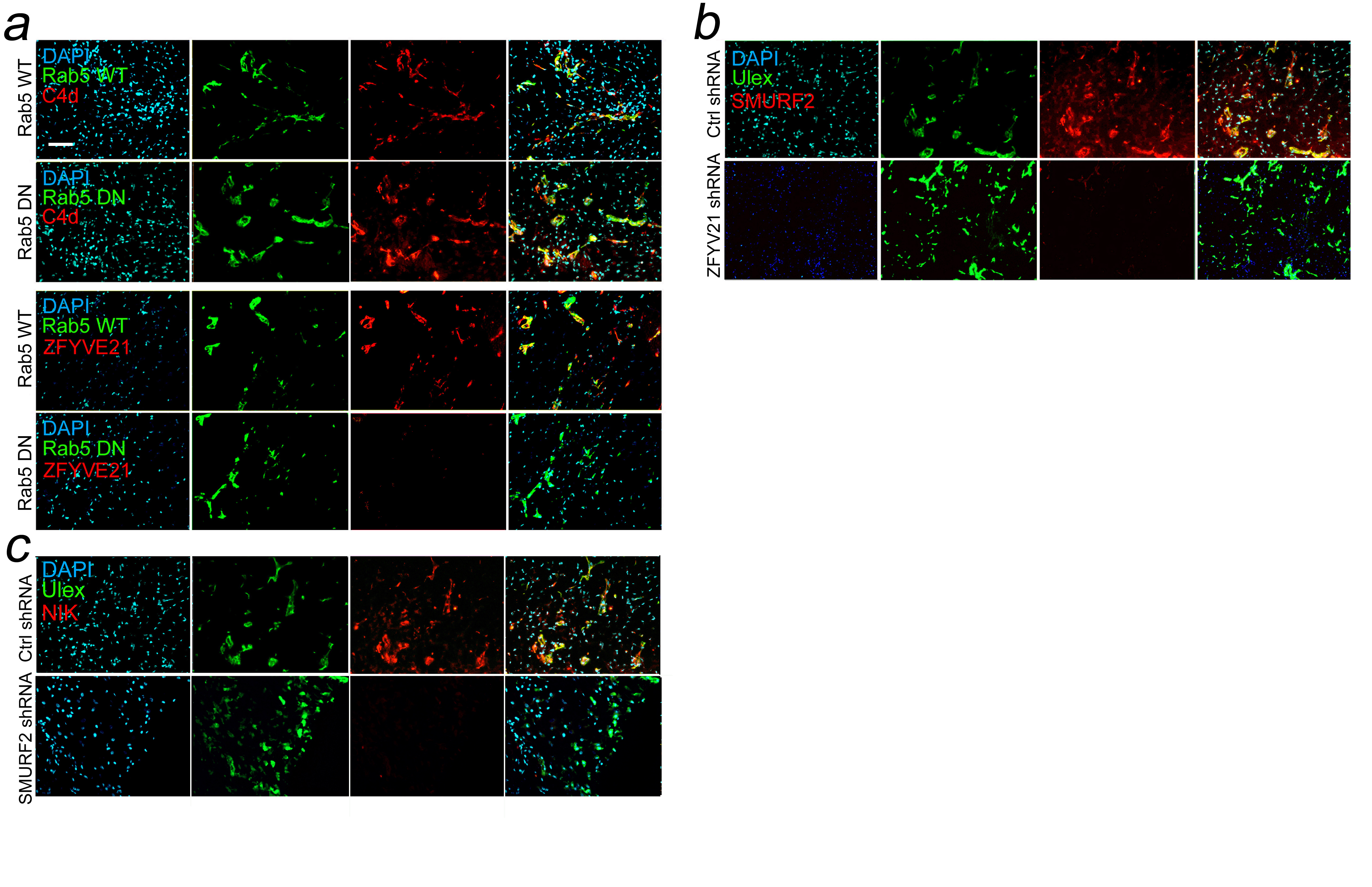
**

**Supplementary Figure 3. The Rab5-ZFYVE21-SMURF2 Signaling Axis Occurs *In Vivo*.** HUVEC stably transduced with Rab5 WT or Rab5 DN (*a*), control or ZFYVE21 shRNA (*b*), or control or SMURF2 shRNA (*c*) were embedded in collagen gel matrices, implanted subcutaneously in SCID/bg immunodeficient mice, and HUVEC-derived microvessels were analyzed 4 weeks later. Twenty-four hours prior to harvest, mice were injected i.v. with 200μL PRA sera. Gels were harvested and stained as indicated (n=3 for all groups, scale bar: 400μm).

**
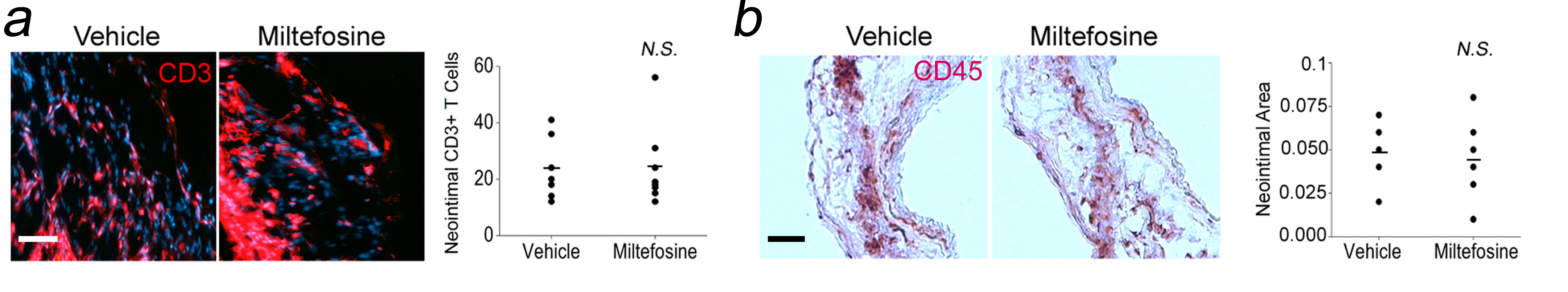
**

**Supplementary Figure 4. Effects of PI(3)P Depletion in the Absence of Complement Activation.** Human coronary artery xenografts were pre-treated with miltefosine (40mg/kg once daily i.p. for 3 days) or vehicle prior to graft harvest and surgical reimplantation as interposition xenografts in the descending aortae of SCID/bg mice engrafted with human T cells. Fourteen days after implantation, human coronary artery segments were harvested and analyzed by I.F. (*a*, scale bar 200μm) and I.H.C. (*b*, scale bar: 800μm).





**Supplementary Figure 5. Western Blot Films Figure 1.** Original uncropped films corresponding to Western blots in Figure 1 in the manuscript.


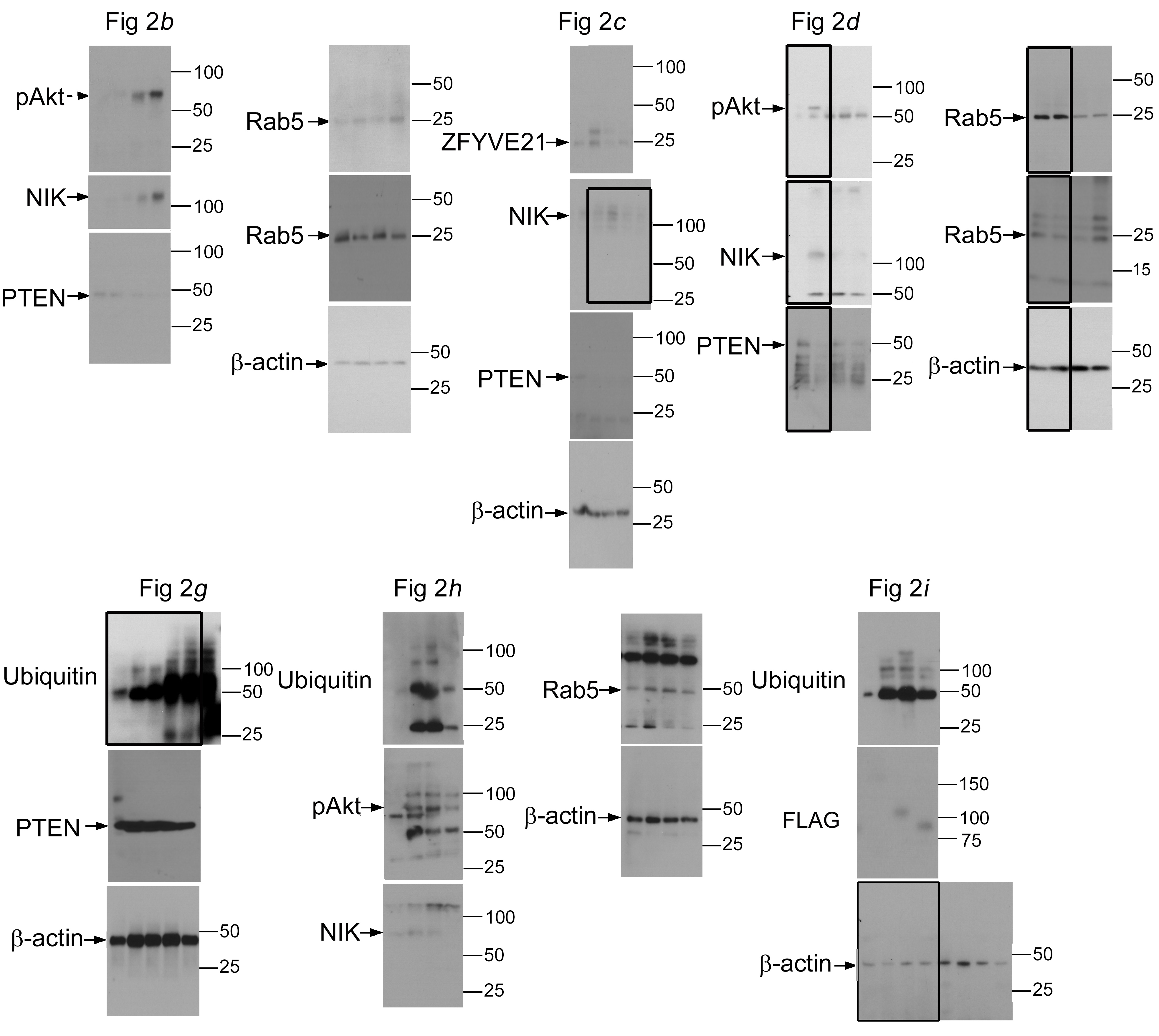


**Supplementary Figure 6. Western Blot Films Figure 2.** Original uncropped films corresponding to Western blots in Figure 2 in the manuscript.


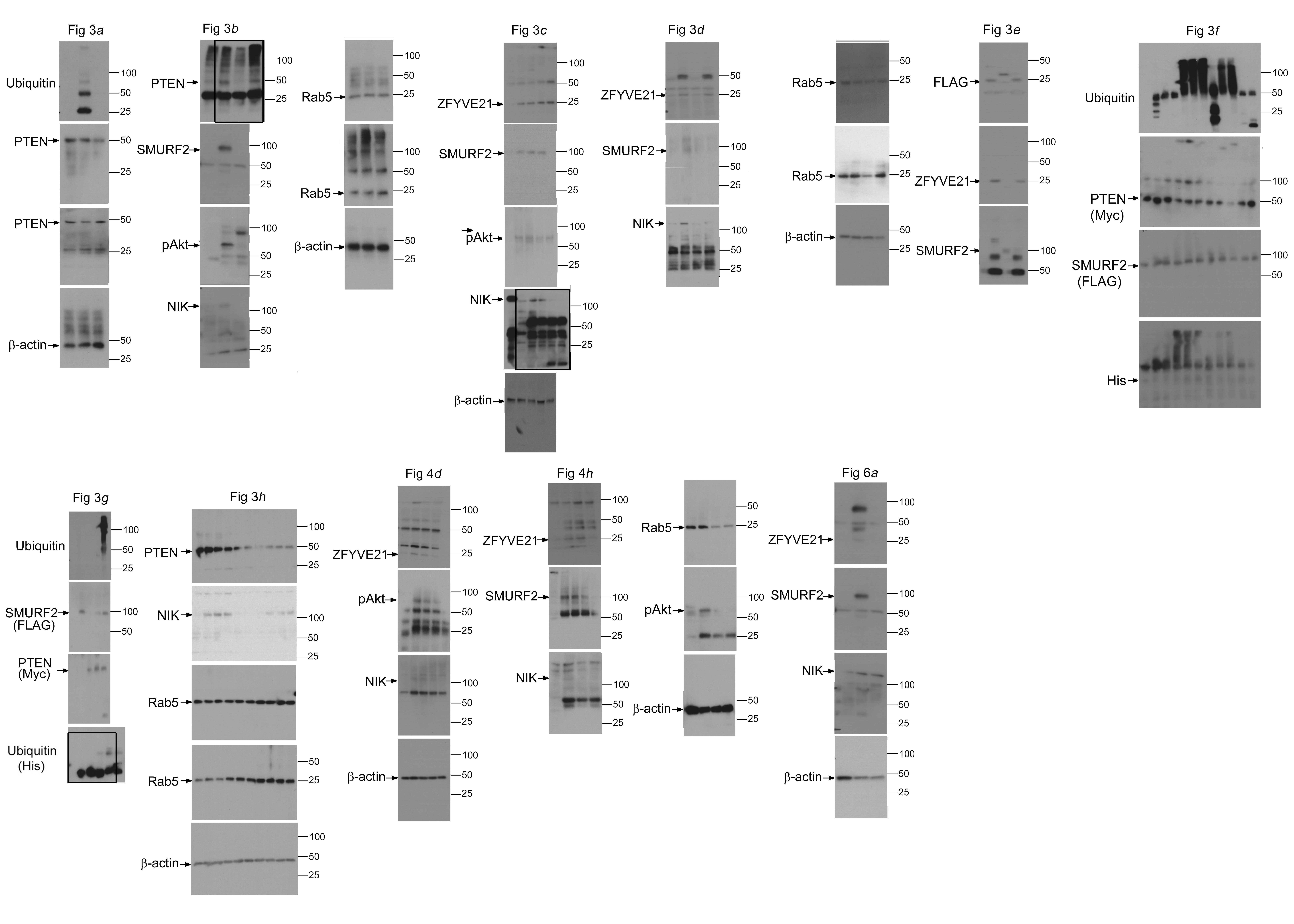


**Supplementary Figure 7. Western Blots Figures 3, 4, and 6.** Original uncropped films corresponding to Western blots in Figures 3, 4, and 6 in the manuscript.

**
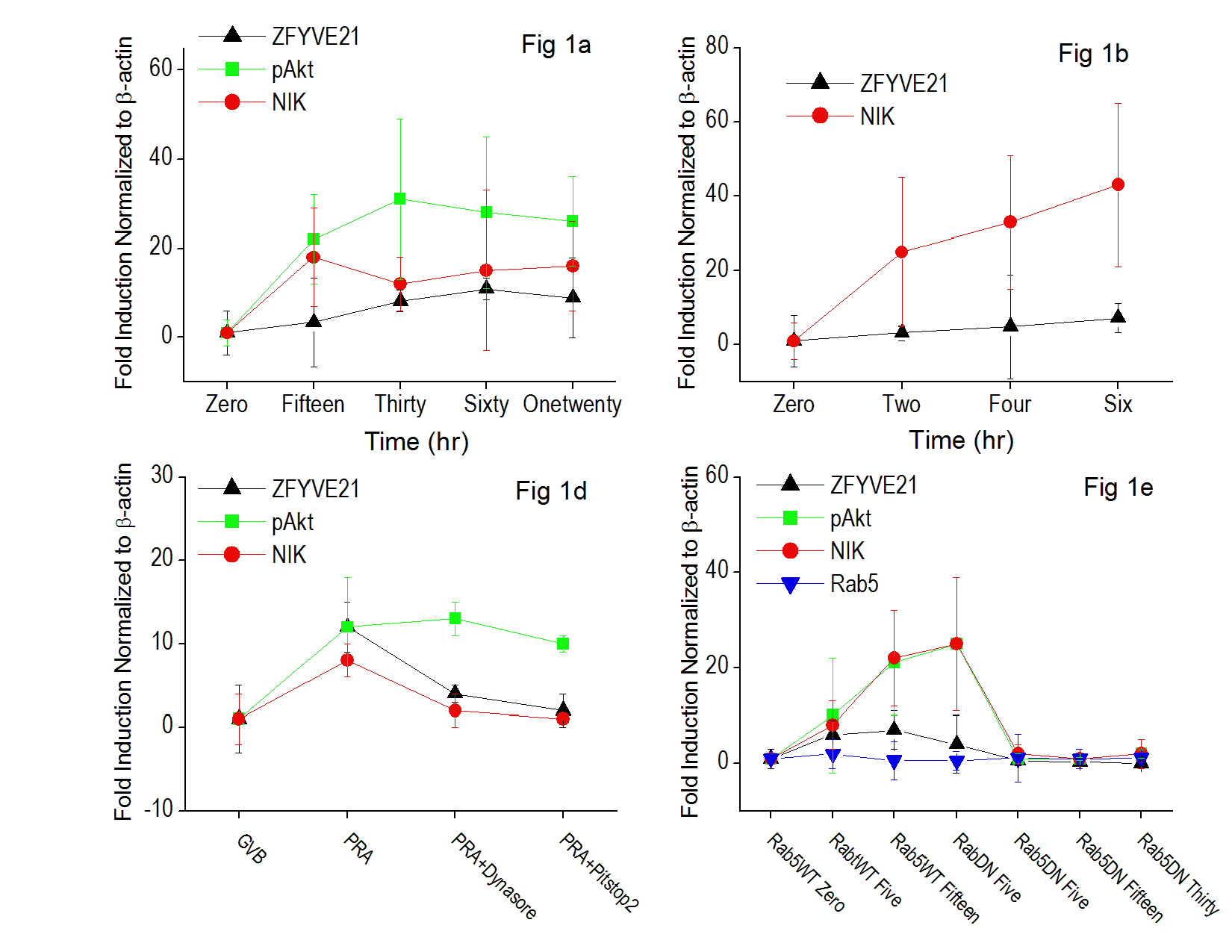
**

**Supplementary Figure 8. Western Blot Quantifications 1.** Densitometric analyses of Western blots in the manuscript for Figures 1*a*, 1*b*, 1*d*, and 1*e*.


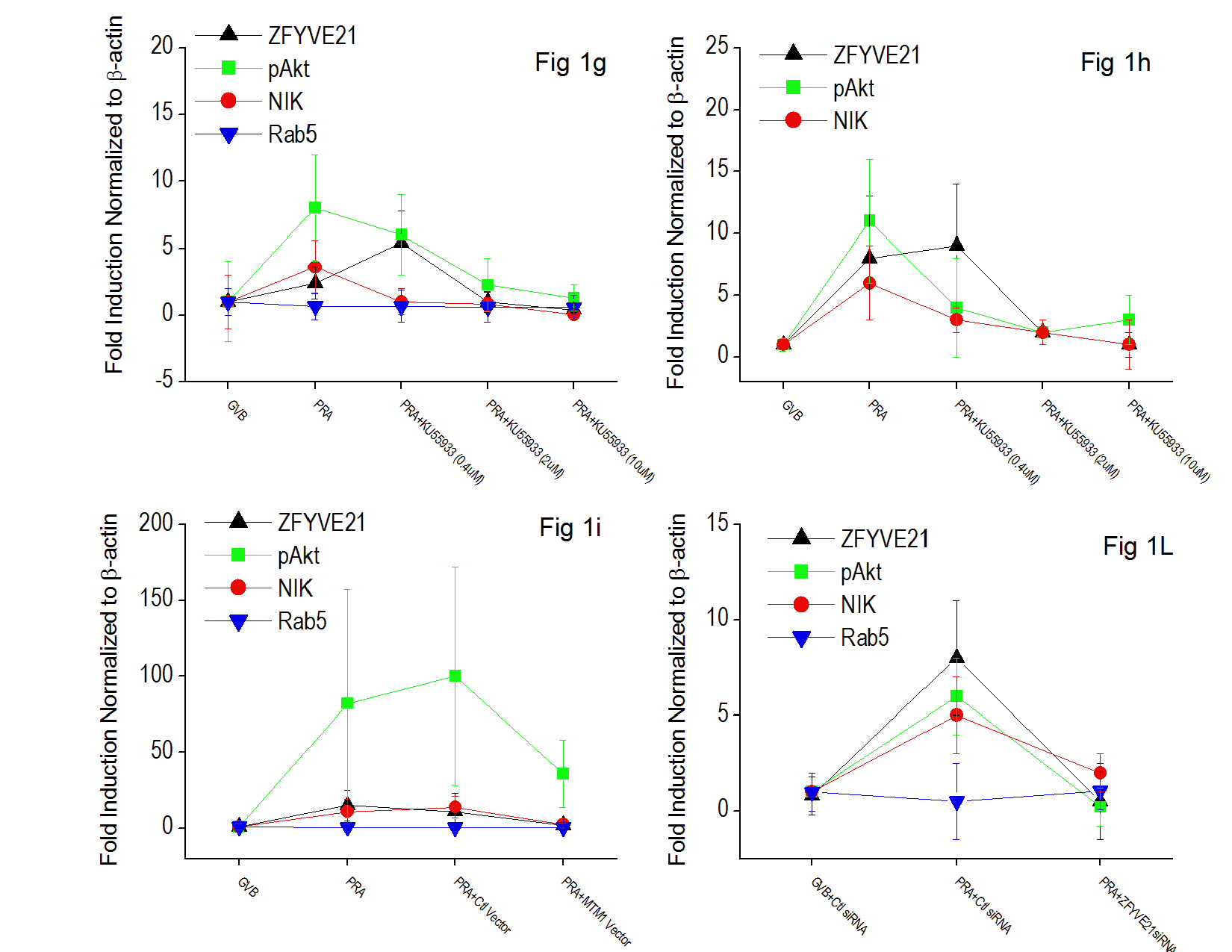


**Supplementary Figure 9. Western Blot Quantifications 2.** Densitometric analyses of Western blots in the manuscript for Figures 1*g*, 1*h*, 1*i*, and 1*l*.


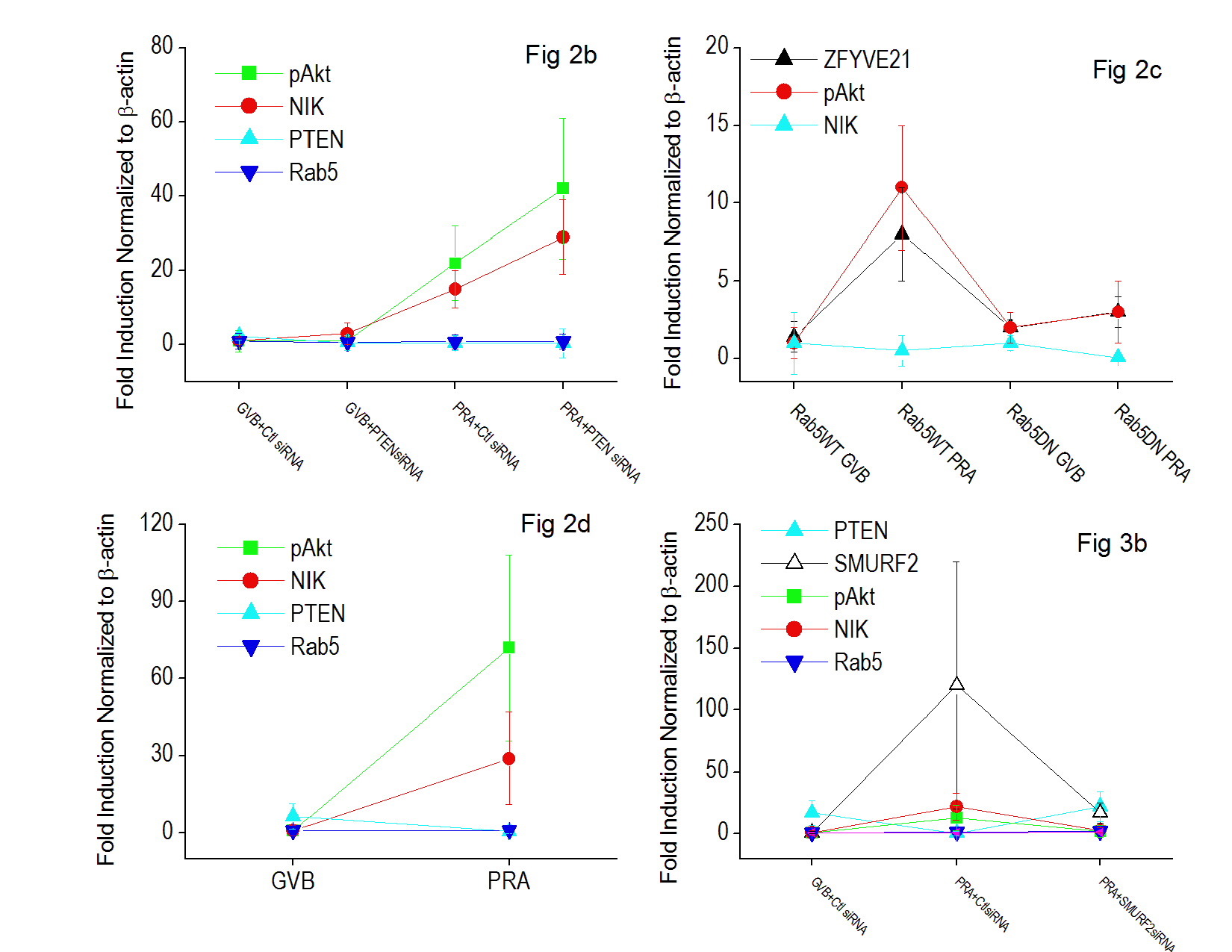


**Supplementary Figure 10. Western Blot Quantifications 3.** Densitometric analyses of Western blots in the manuscript for Figures 2*b*, 2*c*, 2*d*, and 3*b*.


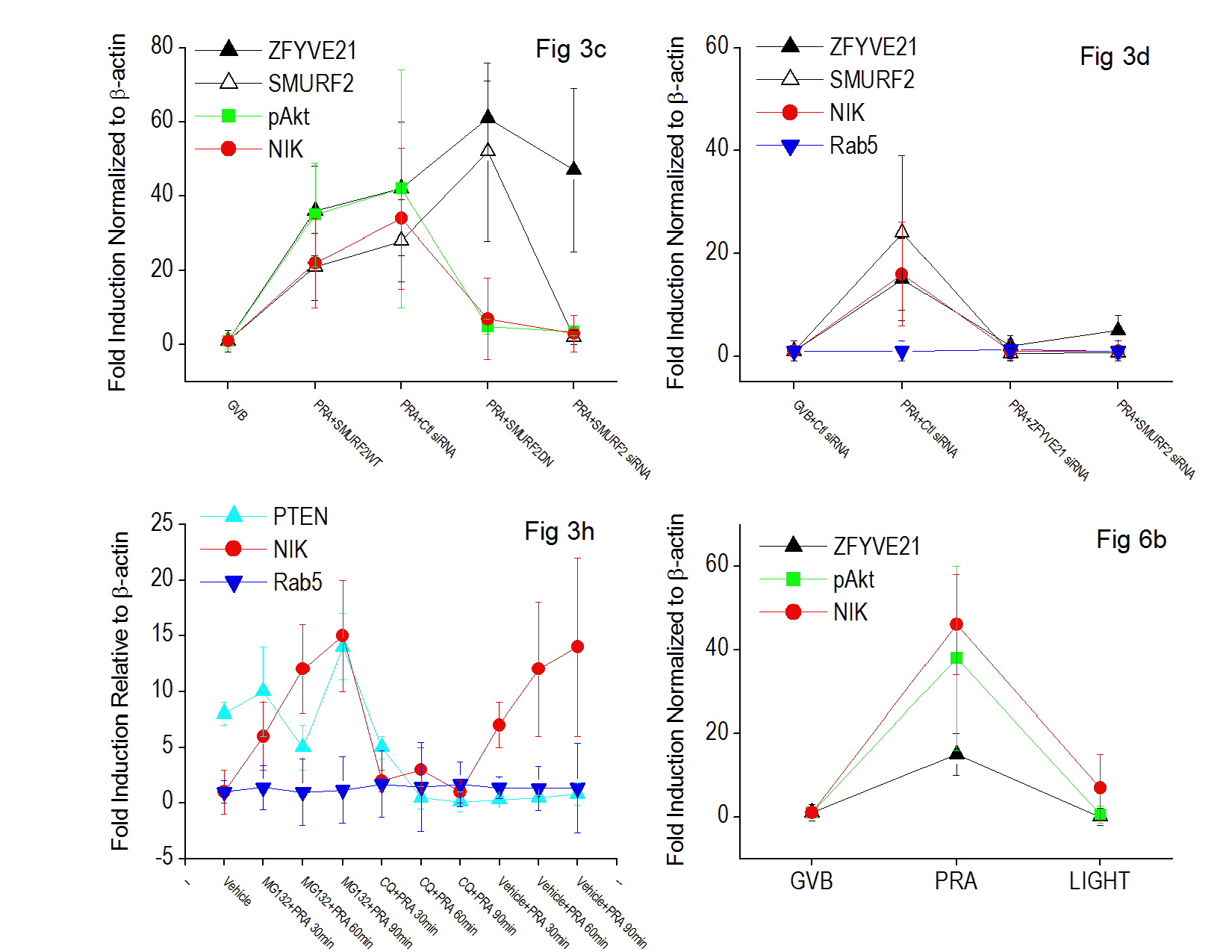


**Supplementary Figure 11. Western Blot Quantifications 4.** Densitometric analyses of Western blots in the manuscript for Figures 3*c*, 3*d*, 3*h*, and 6*b*.
