# Supplementary material for: ZFYVE21 is a complement-induced Rab5 effector that activates non-canonical NF-κB via phosphoinosotide remodeling of endosomes
Source: Nat Commun. 2019 May 21;10:2247. doi: 10.1038/s41467-019-10041-2 (PMC6529429; doi:10.1038/s41467-019-10041-2)
Supplement: Supplementary file 3 — Description of Additional Supplementary Files [file 41467_2019_10041_MOESM3_ESM.docx]

**Description of Additional Supplementary Files**

File Name: Supplementary Data 1

Description: Numerical values for single data points underlying bar graphs in Fig. 1,2,4,6.
