# Supplementary material for: ZFYVE21 is a complement-induced Rab5 effector that activates non-canonical NF-κB via phosphoinosotide remodeling of endosomes
Source: Nat Commun. 2019 May 21;10:2247. doi: 10.1038/s41467-019-10041-2 (PMC6529429; doi:10.1038/s41467-019-10041-2)
Supplement: Supplementary file 5 — Reporting Summary [file 41467_2019_10041_MOESM5_ESM.pdf]

nature research

Corresponding author(s): Dan Jane-wit

Last updated by author(s): Feb 25, 2019

Reporting Summary

Nature Research wishes to improve the reproducibility of the work that we publish. This form provides structure for consistency and transparency in reporting. For further information on Nature Research policies, see Authors & Referees and the Editorial Policy Checklist.

Statistics

For all statistical analyses, confirm that the following items are present in the figure legend, table legend, main text, or Methods section.

n/a	Confirmed	
☐	☒	The exact sample size (*n*) for each experimental group/condition, given as a discrete number and unit of measurement
☐	☒	A statement on whether measurements were taken from distinct samples or whether the same sample was measured repeatedly
☐	☒	The statistical test(s) used AND whether they are one- or two-sided<br>*Only common tests should be described solely by name; describe more complex techniques in the Methods section.*
☒	☐	A description of all covariates tested
☒	☐	A description of any assumptions or corrections, such as tests of normality and adjustment for multiple comparisons
☐	☒	A full description of the statistical parameters including central tendency (e.g. means) or other basic estimates (e.g. regression coefficient) AND variation (e.g. standard deviation) or associated estimates of uncertainty (e.g. confidence intervals)
☒	☐	For null hypothesis testing, the test statistic (e.g. *F*, *t*, *r*) with confidence intervals, effect sizes, degrees of freedom and *P* value noted<br>*Give P values as exact values whenever suitable.*
☒	☐	For Bayesian analysis, information on the choice of priors and Markov chain Monte Carlo settings
☒	☐	For hierarchical and complex designs, identification of the appropriate level for tests and full reporting of outcomes
☐	☒	Estimates of effect sizes (e.g. Cohen's *d*, Pearson's *r*), indicating how they were calculated

Our web collection on statistics for biologists contains articles on many of the points above.

Software and code

Policy information about availability of computer code

Data collection	N/A
Data analysis	N/A

For manuscripts utilizing custom algorithms or software that are central to the research but not yet described in published literature, software must be made available to editors/reviewers. We strongly encourage code deposition in a community repository (e.g. GitHub). See the Nature Research guidelines for submitting code & software for further information.

Data

Policy information about availability of data

All manuscripts must include a data availability statement. This statement should provide the following information, where applicable:

- Accession codes, unique identifiers, or web links for publicly available datasets
- A list of figures that have associated raw data
- A description of any restrictions on data availability

All data are available from the authors upon reasonable request. The proteomics datasets have been publicly deposited in the PRIDE Archive (Accession #: PXD013381).

Field-specific reporting

Please select the one below that is the best fit for your research. If you are not sure, read the appropriate sections before making your selection.

☒ Life sciences ☐ Behavioural & social sciences ☐ Ecological, evolutionary & environmental sciences

Life sciences study design

All studies must disclose on these points even when the disclosure is negative.

Sample size	Sample sizes were determined based on power calculations from pilot studies.
Data exclusions	Data were not excluded from the analysis.
Replication	All experiments were repeated at least twice using two human tissue source donors.
Randomization	Mice were assigned randomly to treated and control groups.
Blinding	Investigators were not blinded to the studies due to the inability to administer relevant treatments to the correct experimental group if blinded.

Reporting for specific materials, systems and methods

We require information from authors about some types of materials, experimental systems and methods used in many studies. Here, indicate whether each material, system or method listed is relevant to your study. If you are not sure if a list item applies to your research, read the appropriate section before selecting a response.

Materials & experimental systems

n/a	Involved in the study	
☐	☒	Antibodies
☒	☐	Eukaryotic cell lines
☒	☐	Palaeontology
☐	☒	Animals and other organisms
☒	☐	Human research participants
☒	☐	Clinical data

Methods

n/a	Involved in the study	
☒	☐	ChIP-seq
☐	☒	Flow cytometry
☒	☐	MRI-based neuroimaging

Antibodies

Antibodies used	Antibodies used for Western blotting were all used at 1:1000 dilution and included ZFYVE21 (Novus Biologicals, #H00079038-B01P), SMURF2 (Cell Signaling Technology, #12024), NIK (Cell Signaling, #4994), PTEN (Cell Signaling, #9188), pAktThr308 (Cell Signaling, #13038), active Rab5 (NewEast Biosciences, #26911), FLAG (Cell Signaling, #14793), Rab5 (Santa Cruz Biotechnology, #sc-46692), K46-conjugated ubiquitin (Cell Signaling, #3936), and ?-actin (Sigma, #A5316-100?L). For immunostaining, stained with relB (Santa Cruz, #sc-226), PI(3)P (Echelon, #Z-P003), PI(3,4,5)P3 (Echelon, #Z-P345b), Sc5b-9 (Dako, M0777), PTEN (Cell Signaling #9188), NIK (Cell Signaling, #4994), and ZFYVE21 (Atlas, HPA055721) at 1:200 dilution for 4oC overnight.
Validation	All primary antibodies were selected based on the manufacturers' validation and prior validations from other groups as reported in the literature.

Animals and other organisms

Policy information about studies involving animals; ARRIVE guidelines recommended for reporting animal research

Laboratory animals	Female SCID/bg mice 6-8 weeks old were used in the study.
Wild animals	N/A
Field-collected samples	N/A
Ethics oversight	All protocols were approved by the Yale IACUC committee.

Note that full information on the approval of the study protocol must also be provided in the manuscript.

Flow Cytometry

Plots

Confirm that:

☒ The axis labels state the marker and fluorochrome used (e.g. CD4-FITC).

☒ The axis scales are clearly visible. Include numbers along axes only for bottom left plot of group (a 'group' is an analysis of identical markers).

☒ All plots are contour plots with outliers or pseudocolor plots.

☒ A numerical value for number of cells or percentage (with statistics) is provided.

Methodology

Sample preparation	Sample preparation for flow cytometry experiments are described in the Methods.
Instrument	The instrument used for FACS analyses (LSRII, Becton Dickinson) is described in the Methods.
Software	Flow-Jo software was used for analysis of flow cytometry experiments.
Cell population abundance	The relevant cell populations for FACS analyses ranged bteween 5-35%. The purity of this population was based on endothelial cells transduced with GFP or RFP.
Gating strategy	The relevant gating strategies for all flow cytometry estudies are shown in the supplemental figures.

☒ Tick this box to confirm that a figure exemplifying the gating strategy is provided in the Supplementary Information.
